# The Association Between Urbanisation and Household Food Security in Nigeria

**DOI:** 10.1111/mcn.70044

**Published:** 2025-05-30

**Authors:** Joseph B. Ajefu, Michael Henry, Sabastine U. Ugbaje

**Affiliations:** ^1^ Department of Peace Studies and International Development University of Bradford Bradford West Yorkshire UK; ^2^ Centre for Social Development in Africa (CSDA) University of Johannesburg Johannesburg Gauteng South Africa; ^3^ Department of Economics, Birmingham Business School University of Birmingham Birmingham West Midlands UK; ^4^ Sydney Informatics Hub, Moore College (CG2) The University of Sydney Newton New South Wales Australia

**Keywords:** association, food security, household, urbanisation

## Abstract

A growing number of studies have shown that urbanisation is commonly associated with a change in dietary or consumption patterns towards more expensive and exotic foods. Previous attempts to investigate the implications of urbanisation on household food security have commonly employed dichotomous or binary indicators of urbanisation. Unlike previous studies, we employ satellite‐based night‐time light intensity data from the US Air Force Defense Meteorological Satellites Programme and use it as a proxy for different stages or degrees of urbanisation. The night‐time light data provide a continuous, spatially explicit, and objective proxy for urbanisation. The data are measured with consistent quality across countries, regardless of the different institutional capacities, allowing for consistent measurement of urban growth across various communities and regions. In our analysis, we explore the impact of variations in nightlight intensity on household food security in Nigeria. Our results show that night‐time light is positively associated with household food security. However, we find that higher polynomial terms of night‐time light intensity exhibit a nonlinear relationship between urbanisation and household food security. Based on the results, our findings will advance the current understanding of the relationship between urbanisation and household food security, which could have implications on maternal and child wellbeing.

## Summary

- Urbanisation could be associated with a change in household consumption or dietary.
- Unlike previous studies that explore binary indicators of urbanisation, as a proxy for urbanisation, this paper uses satellite‐based night‐time light intensity from the US Air Force Defense Meteorological Satellites Programme.
- The findings of the paper reveal that night‐time light is positively associated with household food security, but higher polynomial terms of night‐time light intensity exhibit a nonlinear relationship urbanisation and household food security.

## Graphical abstract

This paper focuses on the nexus between urbanisation and household food security in the context of Nigeria. The study uses night‐time light intensity as a proxy for urbanisation to capture the variation in urbanisation to estimate its relationship to household food security.

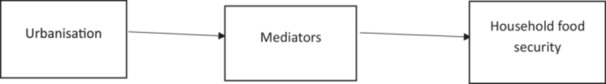

## 1 Introduction

**Figure 1:**
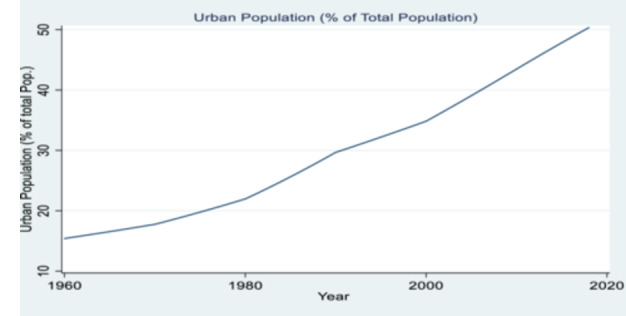
Urban Population (% of Total Population) over time for Nigeria. *Source:* Authors' calculation based on data from the World Bank Development Indicators.

Many developing countries are urbanising at a rapid pace, with countries in Africa accounting for some of the highest urban growth rates in the world. On account of this rapid urban growth, the United Nations projects that over the period 2020–2050 Africa would be the fastest urbanising continent (United Nations 2014). For instance, Nigeria, which is the focus of this study, has witnessed an increase in the proportion of the urban population from 15.41% in 1960% to 50.34% in 2018, with an annual growth rate of the urban population of about 4%–5% over the last 30 years (World Development Indicators 2018, See Figure 1). Figure 1 reveals that urban population (% of total population) in Nigeria has increased steadily over the years.

The rapid growth or expansion of cities and towns (urbanisation)^1^ in low‐ and middle‐income countries such as Nigeria poses rather ambiguous prospects for urban dwellers. Rapid urbanisation in low‐ and middle‐income countries can be associated with urban poverty, youth unemployment, inadequate or low‐quality of physical infrastructure and failure to meet basic services for the growing population (African Development Bank 2011; Ravallion et al. 2007; Dorosh and Thurlow 2014). Conversely, urbanisation may as well provide opportunities for urban residents from the gains associated with urban growth, ranging from industrial and nonfarm employment opportunities to improved market access, high‐value agricultural products and remittances to support rural livelihoods (Bloom et al. 2008; Henderson 2010; Calì and Menon 2013; Vandercasteelen et al. 2018).

A growing number of studies established a link between urbanisation and changes in household dietary intake or consumption (Popkin 1999; Cockx et al. 2018; Colozza and Avendano 2019). In other words, with urbanisation, households are likely to experience shifts in dietary intake towards expensive, packaged or processed exotic foods. Moreover, urbanisation can also result in malnutrition. On one hand, urban household changes in dietary intake and the consumption of highly processed, fatty foods have been strongly linked with increasing health burdens such as obesity (Monda et al. 2007; Popkin et al. 2012; Popkin 2014). Despite the evidence of the positive association between urbanisation and dietary changes, low income of urban dwellers is commonplace among those living in informal settlements or slums in urban areas. Moreover, a lack of income can exacerbate undernutrition or food insecurity, which is the absence of safe and nutritious foods (Cohen and Garrett 2010; Tacoli et al. 2013; Tschirley et al. 2013).^2^

However, empirical evidence on the implications of urbanisation on household food security taking into cognisance the intensity of urbanisation and heterogeneity of urban areas has to a large extent received limited attention in the existing literature. In this paper, we examine the implications of urbanisation on household food security in Nigeria, using the satellite‐based night‐time light intensity data as a proxy for urbanisation intensity and dynamics as well as the associated economic activities.^3^

The data on night‐time lights used in this paper was matched with the Wave 1 (2010/11), Wave 2 (2012/13), Wave 3 (2015/16) and Wave 4 (2018/19) geo‐referenced waves of the Nigerian General Household Survey (GHS) panel data, which enabled us to explore the variation in urbanisation intensity across the enumeration areas over time. Quite unlike the previous studies that used urban population or rural‐urban binary indicators of urbanisation, and which ignored heterogeneities across urban areas (Henderson and Wang 2007; Cockx et al. 2018; Colozza and Avendano 2019), the use of night‐time light data in our analysis provides continuous, spatially explicit, disaggregated, and objective proxy for urbanisation and related economic activities.^4^

This paper contributes to the literature on urbanisation and household food security by investigating explicitly the implications of urbanisation on household food security based on night‐time light intensity as a measure for urbanisation and urban expansion. This approach differs from previous studies that proxied urbanisation by exploring urban areas in terms of small towns, major cities, and urban population (Christiansen and Kanbur 2017; Satterthwaite 2009; Calì and Menon 2013). The night‐time light intensity data provide a continuous measure of urbanisation, which allows our analysis to capture micro‐level variations due to urbanisation across the urban spectrum over time. The intensity of the night‐time lights increases from rural to urban areas (Henderson et al. 2003; Sutton et al. 2010; Donaldson and Storeygard 2016). Further, we identify heterogeneous effects between rural‐urban location and the nexus between urbanisation and household food security in Nigeria.

The paper focuses on Nigeria because of the twin concerns of food insecurity and the expansion of the urban population (Enete and Achike 2008; Owoo 2021); hence, it provides an interesting context to examine the implications of rapid urbanisation on household food security. Over the years, there has been growth in several urban centres in Nigeria, and this reflects the pace of urban growth in the country. Consequently, the growth of towns and cities in Nigeria has been phenomenal due to urbanisation and the rate of urban growth is about 5%–10% per annum (Aliyu and Amadu 2017). It has been argued that the rapid pace of urbanisation in Nigeria results largely from factors such as the discovery of crude oil which has led to an increase in urban wages and a decline in agricultural investment; an increase in the number of states and administrative capitals; poor infrastructure; and lack of social amenities in the rural areas such as roads, electricity and healthcare facilities (see Sackey et al. 2012; Oyeleye 2013).

The relevance of this study is underscored in the second and eleventh agenda of the United Nations Sustainable Development Goals (SDGs), which are: to end hunger, achieve food security and make cities and communities safe, resilient and sustainable by 2030. Recently, evidence revealed that the number of people who face hunger has been on the increase over the years. The statistics show that 122 million more people faced hunger in 2022 than in 2019, pre to the COVID‐19 pandemic. Specifically, the number of people who faced hunger is estimated to be between 691 and 783 million people. Thus, this evidence highlights the imperativeness of this study (see Roser and Ortiz‐Ospina 2018; FAO 2019; FAO et al. 2023).

The paper is broadly at the intersection of two strands of literature: first, it relates to the literature on urbanisation, and its connection with urban and rural poverty. A number of studies support the assertion that migration from rural areas into secondary towns can have a significant effect on poverty reduction compared to big cities. Also, urbanisation can have substantial poverty‐reducing effects in the surrounding rural areas (Christensen and McCord 2016; Calì and Menon 2013; Ingelaere et al. 2018; Christiaensen and Todo 2014). Second, this paper is closely related to studies on urbanisation, child and maternal health (Amare et al. 2020; Abay and Amare 2018; Gong et al. 2012; Goryakin and Suhrcke 2014; Hirvonen 2016; Vlahov 2002). Amare et al. (2020), and Abay and Amare (2018) used night‐time light intensity data to investigate the effect of urbanisation on child nutrition and women's body weight. These studies provide evidence in support of nightlight intensity as a significant predictor of a child's nutritional outcomes and women's weight and obesity.

The remainder of the paper proceeds as follows: Section 2 describes the data sources used in our analysis and presents the summary statistics; Section 3 presents the empirical strategy of the study; Section 4 presents the results of the regression analysis and discussions related to them; Section 5 concludes the paper.

## 2 Data Sources and Summary Statistics

### 2.1 Household Data and Night‐Time Light Intensity Data

This study uses data matched from two sources: the Nigerian General Household Surveys ‐Panel (hereafter, GHS‐Panel) data, and the night‐time light intensity data. We used data from four waves of the Nigerian GHS ‐ Panel data: Wave 1 (2010/11), Wave 2 (2012/13), Wave 3 (2015/16) and Wave 4 (2018/19). We used a balanced panel of households across the four waves of the GHS. The GHS‐Panel is part of the Living Standard Measurement Study (LSMS) ‐Integrated Surveys on Agriculture (ISA) project of the World Bank. The GHS‐Panel Surveys were implemented by the Nigeria National Bureau of Statistics (NBS) with support from the World Bank.

The Nigerian GHS data provide detailed information on individuals, households, and the community over time. It contains demographic and socioeconomic information such as age, education level, household size, gender, marital status, labour market activities, food and nonfood expenditure, indicators of food security, household location (rural/urban), distance to the nearest market and roads etc. The detailed information on household food expenditure and food security indicators allows for different measures of food security such as food access, food availability and food utilisation to be used in our analysis. Further, as the GHS data provides information on the location of each household and their coordinates (latitude and longitude), we were able to match the GHS data with the night‐time light intensity data.

To investigate the relationship between urbanisation and food security, we used satellite‐based night‐time light intensity data obtained from the US Air Force Defense Meteorological Satellites Programme (DMSP) as a proxy for urbanisation or urban growth.^5^ The choice of night‐time light intensity data to measure urbanisation is based on the increasing and credible evidence of night‐time light data capturing micro‐level variations in the rapid dynamics of urban expansion (Amaral et al. 2006). The satellite‐based night‐time light intensity data has received considerable attention because of its potential to capture the dynamics of urbanisation and related economic activities. This stems from the ability to utilise the night‐time data collected via satellite to delineate the spatial extents of urban areas and analyse spatial patterns of both urban and economic growth (Henderson et al. 2011, 2012; Nordhaus and Chen 2015).

Moreover, the satellite‐based night‐time luminosity data relies on access to electricity, and, in most developing countries, electricity is one of the keys to urban amenities, hence, urban areas are expected to have higher night‐time light intensities than rural areas. In view of this, satellite‐based night‐time light luminosity data has been commonly used as a marker of urbanisation (Elvidge et al. 1997; Imhoff et al. 1997; Henderson et al. 2003; Sutton et al. 2010; Zhang and Seto 2011; Amare et al. 2020; Abay and Amare 2018). The data are collected by the DMSP of the United States Air Force Weather Agency at a daily time‐step and at 30 arc‐seconds spatial resolution (~1 km). The light intensity generated is measured in digital numbers, and for each 30 arc‐second pixels, it ranges from 0 (no light) to 63 (highest light). Although the night‐time data is collected by US Air Force Weather Agency at daily time‐step, we obtained annual composites of the stable night‐time light intensity (version 4) data from the portal of the Earth Observation Group, Payne Institute for Public Policy, Colorado School of Mines.^6^ It is imperative to clarify that, though night‐time light is affected by cloud cover, the night‐time light data used in this study is analysis‐ready, meaning it has been pre‐processed to mask pixels with unreliable night‐time light due to factors like cloud‐cover, sunlit and moonlit illumination, and ephemeral events such as fires, and background noise. Thus, we did not perform any additional preprocessing steps on the datasets.^7^

We obtained yearly image composites (one image per year) for the years 2010–2011, 2012–2013, 2015–2016, and 2018–2019 for our analysis.^8^ Because the household survey data we obtained always straddles two calendar years (e.g., 2010 and 2011), we averaged the annual night‐time values for the corresponding calendar years (e.g., average of annual night‐time values for 2010 and 2011). Thus, we averaged night‐time light for each of the survey waves.

Using the GHS GPS coordinates, we extracted the night‐time data for the corresponding survey period, and these were then merged with the GHS datasets. However, we note that the GHS data GPS coordinates were modified by the data providers to maintain the confidentiality of the sampled households and communities. The modification involved adding a random offset to the average coordinates of the households within an enumeration area (EA). In urban areas, an offset of 0–2 km was used, whereas in rural areas an offset of 0–5 km was used for 99% of the data and 0–10 km for the remaining 1% of the data. Nonetheless, the offset points were constrained to the state administrative boundary so that points do not fall outside the state where the EA is located.

**Figure 2:**
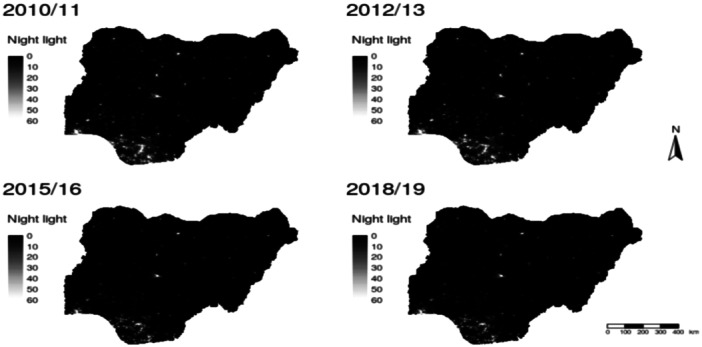
Night‐time light intensity at districts for 2010/11, 2012/13, 2015/16, and 2018/19. *Source:* Image and data processing by NOAA's National Geophysical Data Centre. DMSP data was collected by the United States Air Force Weather Agency.

To account for the GPS modification in our data extraction procedure, we extracted an averaged value from pixels within a 1, 2, 4, 5, and 10 km buffer zones of the modified GPS points. Subsequently, we modelled and evaluated the relationship between each set of night‐time data extracted within the buffers and the GHS data and report only results from the best model. For brevity, however, we did not evaluate separate models for the rural or urban areas. Figure 2 shows the geographic distribution of night‐time lights across Nigeria for years corresponding to the four waves of data used in our analysis. Generally, the night‐time lights spatial density increases as we move from the northern region to the southern region in Nigeria. From Figure 2, the night‐time lights range from low night‐time lights which are captured by the dark spot (darkness) on the maps to high night‐time lights represented by bright spots on the maps.

**Figure 3:**
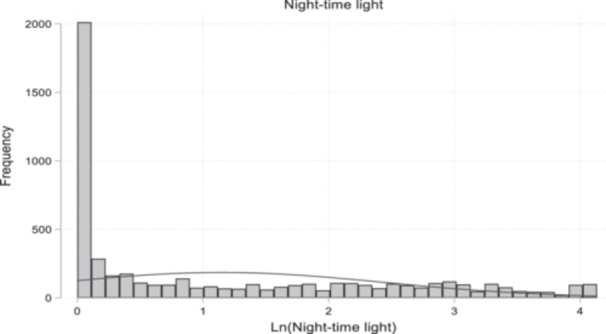
Histogram and normal density of night‐time light intensity in Nigeria using data from the NOAA's National Geophysical Data Centre.

**Figure 4:**
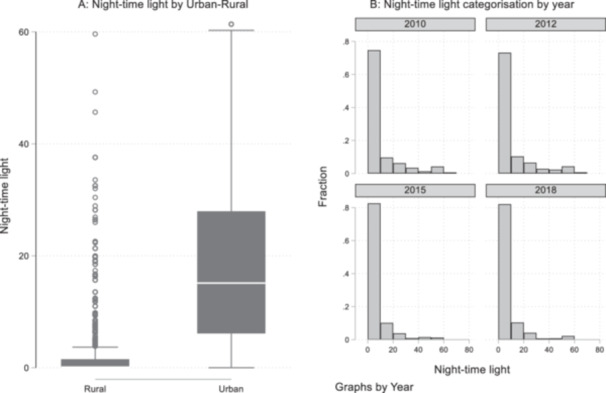
(A) Box and Whisker Plot for night‐time light intensity by urban‐rural classification; and (B) Distribution of night‐time light intensity by year, between 2010 and 2018. *Source:* NOAA's National Geophysical Data Center.

Figure 3 shows the histogram and normal density of night‐time light intensity across the four waves—between 2010/11 and 2018/2019. We used the logarithm of night‐time light intensity for the plot. The distribution of night‐time lights ranges from 0 to 63 digital numbers, while the logarithm of night‐time light ranges from 0 to 4. The histogram shows the concentration of night‐time lights around 0 and 1 of the log‐transformed digital number, which denotes low night‐time light intensity. Figure 4A shows a Box and Whisker plot for night‐time light intensity by urban‐rural classification, and Figure 4B shows the distribution of night‐time light intensity by year from 2010 to 2018. Figure 4A shows that, not unsurprisingly, night‐time lights are higher in urban areas compared to rural areas. From Figure 4B, the fraction of low intensity light increased in 2015 and 2018 compared to 2010 and 2012.

### 2.2 Household Food Security Indicators

We adopt a comprehensive approach to measuring household food security outcomes. As proposed in the literature, this approach follows a multi‐dimensional food security definition, which focuses on food security metrics or indicators (Upton et al. 2016; Brück et al. 2019; Dedehouanou and McPeak 2020; Rahman and Mishra 2020; Amolegbe et al. 2021). First, we used the household dietary diversity score, which is the number of different food group categories consumed by the households in the last 7 days before the survey (Efobi et al. 2020; Ajefu and Abiona 2020; Ajefu et al. 2021; Ajefu et al. 2024). We computed the household dietary diversity score for 11 food categories, with the maximum score for a household being 11 and minimum score of zero. Second, following the World Food Programme (WFP) guidelines, we calculated the food consumption score. This variable (or measure) is the weighted sum of the number of days the household consumed foods from eight food groups in the last 7 days before the survey.^9^

Third, we computed the Household Food Insecurity Access Scale (HFIAS) score using the 2010, 2012, 2015 waves of the Nigerian GHS. We are unable to use the 2018 wave because the survey does not provide consistent information with other waves of the GHS to generate the HFIAS score. To compute the HFIAS, we use information captured in Nigerian GHS, which indicates that, in the past 7 days, how many days have you or someone in your household had to: (a) Rely on less preferred foods? (b) Limit the variety of foods eaten? (c) Limit portion size at mealtimes? (d) Reduce number of meals eaten in a day? (e) Restrict consumption by adults for small children to eat? (f) Borrow food, or rely on help from a friend or relative? (g) Have no food of any kind in your household? (h) Go to sleep at night hungry because there is not enough food? (i) Go a whole day and night without eating anything? Using the HFIAS Indicator Guide, v.2, we coded the responses into the following: rarely = 1 (0–1 days), sometimes = 2 (2–3 days), and often = 3 (4–7 days). The maximum number for the sum of different responses to compute the HFIAS is 27. It is important to note that a higher HFIAS indicates higher levels of food insecurity.

Fourth, we computed a disaggregated measure of household consumption of each food category or class using the information on the number of days in a week as captured by the Nigerian GHS across the four waves. Hence, we compute the following: the average number of days households consume grains flour, roots and tubers, pulses and nuts, vegetables, meats, fruits, milk, oil/fats, and sugar.

### 2.3 Summary Statistics

Table 1 presents the descriptive statistics of the variables used in our analysis across the four waves of the Nigerian GHS. The average food consumption score increased from 55.4 in wave 1 to 58.5 in wave 4 out of a maximum score of 126. The average household dietary diversity score increased from 7.8 in wave 1 to 8.7 in wave 4 out of a maximum score of 11, and the HFIAS score increased from 9.419 in wave 1 to 10.724 in wave 3 In this study, as discussed in the previous section, we proxied urbanisation by using night‐time light intensity which is reported as a digital number, and it is an integer that ranges from 0 to 63. The descriptive statistics reported a decrease in night‐time light intensity from 9.005 to 5.209. For household explanatory variables (characteristics) used in the regression, average household size increased from about 6 people in a household to about 8 people in a household across waves 1–4. The average household heads reported in wave 1, about 86% are male, and declined to about 77% in wave 4. The average number of married household heads declined from 82% in wave 1% to 71% in wave 4, and the average age of household heads increased from 50 years in wave 1 to 54 years in wave 4. The percentage of household resident in urban location remain fairly the same across the four waves (29%). In addition to using household control variables, we used community‐level characteristics, which are distance to the nearest road, distance to the nearest market, and the annual rainfall, reported across the four waves.

**Table 1:** Summary statistics of the variables (proportions reported in percentages).

	Wave 1	Wave 2	Wave 3	Wave 4
Variable	Mean (Std dev)	Mean (Std dev)	Mean (Std dev)	Mean (Std dev)
*Dependent variable*				
Food consumption score (FCS)	55.412 (22.810)	56.553 (22.867)	56.701 (21.626)	58.552 (20.650)
HH dietary diversity score (HDDS)	7.889 (2.126)	7.879 (1.964)	7.923 (1.916)	8.780 (1.604)
HFIAS score^10^	9.419 (1.191)	10.516 (2.389)	10.724 (2.378)	N/A
*Other variables*				
Night‐time light intensity	9.005 (14.597)	9.063 (14.722)	5.180 (10.396)	5.209 (10.770)
Household size	5.915 (3.014)	6.442 (3.192)	7.244 (3.507)	7.555 (3.822)
Male head household	86.200 (0.344)	84.600 (0.360)	79.500 (0.403	76.600 (0.423)
Household head married	81.900 (0.384)	78.300 (0.412)	75.800 (0.427)	70.900 (0.454)
Age of household head	49.744 (14.902)	52.164 (14.603)	52.793 (13.916)	54.229 (13.905)
HH at least secondary education	12.800 (0.334)	14.200 (0.348)	15.400 (0.361)	15.700 (0.364)
HH engaged in economic activity	88.500 (0.318	63.200 (0.327)	58.300 (0.380)	50.000 (0.378)
Urban residents	28.500 (0.451)	28.800 (0.453)	28.500 (0.451)	29.300 (0.455)
No. of children below 5 years	1.109 (1.269)	0.940 (1.169)	0.828 (1.124)	0.686 (1.069)
No. of children btw 6 and 14 years	1.516 (1.476)	1.644 (1.594)	1.606 (1.626)	1.671 (1.680)
No. of adults btw 15 and 60 years	2.99 (1.711)	3.261 (1.970)	2.956 (1.894)	3.057 (2.043)
No. of adults above 60 years	0.296 (0.543)	0.367 (0.632)	0.392 (0.610)	0.431 (0.628)
HH dist. to the nearest market (km)	69.963 (46.011)	69.792 (45.961)	70.085 (46.012)	60.677 48.442
HH dist. to the nearest road (km)	13.512 (18.579)	6.254 (8.260)	6.199 (8.260)	5.621 (7.563)
Average annual rainfall (mm)	1269.32 (383.896)	1366.71 (349.363)	1219.038 (403.514)	1432.278 (550.333)
Number of households	1348			
Observations	5392			

*Source:* Authors' calculation based on data from the Nigeria GHS 2010/11, 2012/13, 2015/16, and 2018/19 waves. Standard deviation is in parentheses.

Table 2 presents the mean difference in night‐time light and household food security outcomes between rural and urban households using the four waves of the data. The urban residents reported a food consumption score of 61.965 compared to 54.715 of the rural residents. While the average night‐time light is 2.059 digital numbers for rural areas, the average night‐time light for urban areas is about 19.597 digital numbers. The household dietary diversity was about 7.647 for urban dwellers compared to 6.844 for rural residents. The HFIAS score, on average, is 10.420 for rural residents, compared to 10.194 for urban dwellers. For the disaggregated food categories, on average, except for grains flour and oil/fats, urban residents reported a greater number of days than rural residents that each category of food was consumed. The mean differences between rural and urban dwellers in food categories consumed and the number of days reflect the difference in consumption or food security that exists among households in developing countries. The mean difference measures the absolute difference between the mean value of rural and urban households in relation to the variables in Table 2. The results indicate the *p*‐values for comparison of mean difference (tests) for the variables used in our analysis. The mean tests reveal significant differences between rural and urban residents for a number of the variables.

**Table 2:** Tests of mean difference in urbanisation and food security between urban and rural households.

Variable	Rural (*R*_0_)	Urban (*R*_1_)	Test = *R*_1_ *− R*_0_
Night‐time lights Food consumption score (FCS)	2.059 (0.079) 54.715 (0.352)	19.597 (0.440) 61.965 (0.549)	17.537*** (0.306) 7.249*** (0.655)
Household dietary diversity Score (HDDS)	6.844 (0.023)	7.647 (0.033)	0.804*** (0.041)
HFIAS score	10.420 (0.039)	10.194 (0.072)	0.226*** (0.077)
No. of days for grains flour No. of days for roots & tubers No. of days for pulses and nuts No. of days for vegetables No. of days for meat & products No. of days for fruits No. of days for milk/products No. of days for oil/fats No. of days for sugar/products	5.057 (0.034) 3.397 (0.039) 2.816 (0.031) 4.954 (0.034) 3.464 (0.038) 1.103 (0.025) 1.373 (0.033) 5.562 (0.032) 2.340 (0.041)	4.774 (0.049) 3.952 (0.052) 2.826 (0.041) 4.981 (0.051) 4.294 (0.057) 1.613 (0.042) 2.088 (0.053) 5.449 (0.051) 2.370 (0.061)	*−*0.282*** (0.062) 0.555*** (0.069) 0.009 (0.056) 0.027 (0.063) 0.829*** (0.070) 0.509*** (0.047) 0.716*** (0.061) *−*0.112* (0.061) 0.031 (0.075)

*Note:* *, **, and *** indicate significance at 10%, 5% and 1% respectively (ii) Standard errors in parentheses.

Authors' calculation based on data from the Nigeria GHS 2010/11, 2012/13, 2015/16, and 2018/19 waves.

### 2.4 Ethics Statement

The study did not require ethical approval because it used secondary data (the Nigeria General Household Surveys) publicly available to researchers. The surveys were conducted in partnership between the National Bureau of Statistics (NBS), the Federal Ministry of Agriculture and Rural Development, the National Food Reserve Agency, the Bill and Melinda Gates Foundation, and the World Bank.

## 3 Empirical Strategy

We explore the four‐period panel data nature of the Nigerian GHS to estimate the relationship between urbanisation and household food security. The use of a household‐level panel data set exploits temporal—within the household, and spatial variations in nightlight intensity by controlling for time‐invariant heterogeneities across households and districts (Abay et al. 2023). Therefore, the use of panel data analysis is likely to mitigate the endogeneity concerns associated with the use of satellite‐based nightlight intensity data as a proxy for urbanisation. The endogeneity could stem from the correlation between urban expansion and economic growth, which can influence livelihoods and household food security status of households.

This estimation approach considers the likelihood of nonlinearities of the household's exposure to night‐time light intensity across the urban spectrum over time. Following Abay and Amare (2018), Mitra and Nagar (2018), and Amare et al. (2024), we capture the possibility of nonlinearities in night‐time light intensity in our analysis by including the second‐order polynomial terms for the night‐time light between urbanisation and household food security, but there could be non‐linearities at high levels of urbanisation. The nonlinearity in the relationship between urbanisation and household food security could stem from households' exposure to negative externalities that affect household food security such as the high cost of land, difficulty to access housing, and high transport costs, among others, as urbanisation reaches a certain threshold limit.

We estimate the relationship between urbanisation and household food security using the equation below:

(1) $${\mathrm{FS}}_{\mathrm{hdt}}=\beta_{0}+\beta_{1}{\ln\mathrm{\_night\_lights}}_{\mathrm{dt}}+\beta_{2}{\left({\ln\mathrm{\_night\_lights}}_{\mathrm{dt}}\right)}^{2}+\beta_{3}X_{\mathrm{hdt}}+\gamma_{r}+\delta_{t}+\varepsilon_{\mathrm{hdt}}$$

where ${\mathrm{FS}}_{\mathrm{hdt}}$ denotes the food security status of household *h* from district *d* at time *t*. We consider the following food security indicator: food consumption score (FCS), household dietary diversity (HDDS), and HFIAS score. The variable ${\ln\mathrm{\_night\_lights}}_{\mathrm{dt}}$ is our variable of interest. It denotes the logarithm of night lights intensity at district *d* for the four‐time periods. It measures the degree of urbanisation across space over time. As stated above, we also include a quadratic term of the logarithmic of night light intensity in the regressions. $X_{\mathrm{hdt}}$ denotes household and community covariates$;\;\gamma_{r},\mathrm{and}\;\delta_{t}$ captures region and time fixed effects, respectively. $\varepsilon_{\mathrm{hdt}}$ is the error term for household *h* in district *d* at time *t*, and standard errors are clustered at the enumeration areas level.

## 4 Results

Using night‐time light intensity as a proxy for urbanisation, Table 3 (columns 1–3), presents results of the quadratic regressions of the association between urbanisation and the different indicators of household food security. The results show that night‐time light intensity is positively associated with both the FCS and the dietary diversity score but negatively associated with HFIAS score (which indicates a decline in food insecurity). Moreover, increasing the average night‐time light intensity by 10% is associated with about a 0.02 decline in the HFIAS score, but an increase in FCS and HDDS of about 0.68 and 0.06, respectively. However, the coefficients of night‐time light intensity squared have negative and statistically significant effects on our food security indicators (except for the HFIAS score), such as FCS and HDDS scores. These findings indicate significant nonlinearity in the relationship (association) between urbanisation (proxied by night‐time light) and household food security status. As robustness check, Table A1 presented in the appendix used urban dummy as the main explanatory variable. The results reveal that urban dummy is positive and statistically significant with both the FCS and the dietary diversity score but negatively associated (though not statistically significant) with the HFIAS score. The result is consistent with the results in Table 3.

**Table 3:** Urbanisation and food security.

	HFIAS score	FCS	HDDS
Variables	(1)	(2)	(3)
Ln (night‐time light)	−0.253* (0.147)	7.122*** (1.736)	0.673*** (0.138)
Ln (night‐time light)‐square	0.055 (0.040)	−1.414*** (0.495)	−0.086** (0.037)
Constant	6.416*** (1.989)	48.185* (27.413)	5.814*** (1.954)
Household control	Yes	Yes	Yes
Community control	Yes	Yes	Yes
Region fixed effect	Yes	Yes	Yes
Year fixed effect	Yes	Yes	Yes
R‐squared	0.207	0.079	0.174
Observations	3852	5392	5392

*Note:* *, **, and *** indicate levels of significance at 10%, 5%, and 1%. Additional control variables used in the regression include household size, household head male, household head married, the household head has a secondary education, Household head engaged in economic activity, no. of children 5 years in the household, no. of children between 6 and 14 years, no. of adults between 15 and 60 years, no. of adults above 60 years, the household distance to the nearest market, the household distance to the nearest road, and average annual rainfall. The regressions used *reghdfe* as the Stata command, and it include regional and year fixed effects. Standard errors clustered at the enumeration area level are given in parentheses.

In Table 4 (Columns 1–9), we present the results of disaggregated food categories or classes consumed by households. We find that night‐time light intensity is positively associated and statistically significant with the consumption of cereals/grains, pulses/nuts, vegetables, meat/products, fats/oil, meat and meat product, and sugar/sugar products, but not statistically significant with roots/tubers. However, we find a negative and statistically significant association between nightlight intensity squared and food classes such as cereals, roots/tubers, pulses, vegetables, meat, fats/oil, but not statistically significant with fruits, milk and sugar products.

**Table 4:** Urbanisation and food security (disaggregated food categories).

	Cereals/grains	Roots/tubers	Pulse/nut	Vegetables	Meat/products	Fruits	Milk & products	Fats/oil	Sugar/products
Variables	(1)	(2)	(3)	(4)	(5)	(6)	(7)	(8)	(9)
Ln (night‐time light)	0.217* (0.121)	0.120 (0.153)	0.334*** (0.119)	0.258** (0.132)	0.623*** (0.159)	0.292** (0.125)	0.511*** (0.171)	0.234* (0.139)	0.492*** (0.167)
Ln (night‐time light)‐square	−0.0662** (0.033)	−0.068* (0.038)	−0.089*** (0.033)	−0.066* (0.040)	−0.139*** (0.043)	−0.044 (0.032)	−0.037 (0.045)	−0.089** (0.040)	−0.046 (0.045)
Household control	Yes	Yes	Yes	Yes	Yes	Yes	Yes	Yes	Yes
Community control	Yes	Yes	Yes	Yes	Yes	Yes	Yes	Yes	Yes
Region fixed effect	Yes	Yes	Yes	Yes	Yes	Yes	Yes	Yes	Yes
Year fixed effect	Yes	Yes	Yes	Yes	Yes	Yes	Yes	Yes	Yes
Constant R‐squared	9.529*** (2.069) 0.309	−7.046*** (0.228) 0.260	3.065* (1.833) 0.081	4.863** (2.363) 0.068	2.661 (2.641) 0.140	1.153 (1.358) 0.098	2.855 (2.418) 0.101	6.412*** (2.375) 0.052	5.474** (2.209) 0.209
Observations	5392	5392	5392	5392	5392	5392	5392	5392	5392

*Note:* *, **, and *** indicate levels of significance at 10%, 5%, and 1%. Additional control variables used in the regression include household size, household head male, household head married, the household head has a secondary education, Household head engaged in economic activity, no. of children 5 years in the household, no. of children between 6 and 14 years, no. of adults between 15 and 60 years, no. of adults above 60 years, the household distance to the nearest market, the household distance to the nearest road, and average annual rainfall. The regressions used *reghdfe* as the Stata command, and it includes regional and year fixed effects. Standard errors clustered at the enumeration area level, are given in parentheses.

### 4.1 Nonparametric Associations Between Night‐Time Lights and Food Security Outcomes

**Figure 5:**
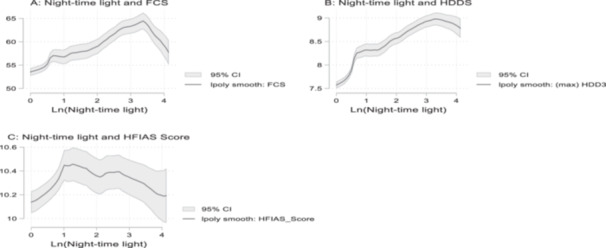
Plots of polynomial between night‐time lights and food security outcomes.

Figure 5 presents nonparametric and unconditional regressions that depict the relationship between urbanisation and food security outcomes. The graphs reveal nonlinearity in the relationship between night‐time light (proxy for urbanisation) and food security outcomes.

Figure 5a, indicates a positive association between night‐time light and food security outcomes (FCS and HDDS), but at the upper end of the night‐time light distribution the shape of the graphs changed to inverse relationship (association). From Figure 5c, at the lower end of the night‐time light distribution there appears to be a positive association between night‐time light and HFIAS score, but at the upper portion of the distribution the graph depicts a negative association between night‐time light and HFIAS score.

### 4.2 Heterogeneous Effects of Urbanisation and Food Security

In this sub‐section, we investigate the heterogeneity of the effect of urbanisation in urban‐rural location and gender of the household head. The choice of these variables stems from the possibility of the variables affecting household food security (Codjoe et al. 2016; Negesse et al. 2020). In Table 5, the results reveal that magnitude of the relationship between degree of urbanisation and food security is larger among urban households compared to rural households, except for HDDS, which is not statistically significant.

**Table 5:** Effects of interaction between urbanisation and urban‐rural dummy.

	HFIAS score	FCS	HDDS
Ln (night‐time light)	−0.206*** (0.074)	3.102*** (1.012)	0.360*** (0.079)
Ln (NL) × urban	−0.175* (0.1231)	2.839** (1.294)	0.067 (0.1038)
Urban dummy	−0.0029 (0.324)	7.758** (2.631)	0.387* (0.233)
Constant	6.517*** (1.960)	49.208* (27.318)	5.857*** (1.917)
Household control variables	Yes	Yes	Yes
Community control variables	Yes	Yes	Yes
Region fixed effect	Yes	Yes	Yes
Year fixed effect	Yes	Yes	Yes
R‐squared	0.210	0.078	0.174
Observations	3852	5392	5392

*Note:* *, **, and *** indicate levels of significance at 10%, 5%, and 1%. Urban dummy = 1 if urban residents or urban dummy = 0 if rural residents. Additional control variables used in the regression include household size, household head male, household head married, the household head has a secondary education, household head engaged in economic activity, no. of children 5 years in the household, no. of children between 6 and 14 years, no. of adults between 15 and 60 years, no. of adults above 60 years, household distance to the nearest market, household distance to the nearest road, and average annual rainfall. The regressions used *reghdfe* as the Stata command, and it include regional and year fixed effects. Standard errors clustered at the enumeration area level, are given in parentheses.

In Table 6, we present the results of the heterogeneous effects of gender of the household head in the relationship between urbanisation and food security.

**Table 6:** Effects of interaction between urbanisation and gender of household head.

	HFIAS score	FCS	HDDS
Ln (night‐time light)	−0.048 (0.106)	2.311*** (0.766)	0.349*** (0.066)
Ln (NL) × male‐headed household	−0.027 (0.092)	0.176 (0.728)	0.048 (0.055)
Male‐headed household	−0.087 (0.245)	−1.748 (1.753)	−0.293* (0.158)
Constant	6.483 (2.020)	47.055* (27.110)	5.807*** (1.922)
Household control variables	Yes	Yes	Yes
Community control variables	Yes	Yes	Yes
Region fixed effect	Yes	Yes	Yes
Year fixed effect	Yes	Yes	Yes
R‐squared	0.207	0.073	0.172
Observations	3852	5392	5392

*Note:* *, **, and *** indicate levels of significance at 10%, 5%, and 1%. Male‐headed household = 1 if household head is male or male‐headed household = 0 if household head is female. Additional control variables used in the regression include household size, household head married, the household head has a secondary education, Household head engaged in economic activity, no. of children 5 years in the household, no. of children between 6 and 14 years, no. of adults between 15 and 60 years, no. of adults above 60 years, the household distance to the nearest market, the household distance to the nearest road, and average annual rainfall. The regressions used *reghdfe* as the Stata command, and it include regional and year fixed effects. Standard errors clustered at the enumeration area level are given in parentheses.

For the interaction term of night‐time light and male‐headed household, we find no statistically significant effects for the measures of food security outcomes used in our analysis.

### 4.3 The Mechanisms of Effects of Urbanisation on Food Security

In this sub‐section, we explore the potential pathways through which urbanisation affects household food security. We identified market access, such as distance to the nearest market, and distance to the nearest road as the mechanisms that linked urbanisation to household food security. From Table 7, using structural equation modelling (SEM) method, the results from Panel A show a direct pathway between urbanisation and food security outcomes, including market access, while the results from Panel B indicate an indirect pathway through which urbanisation affects food security. From the indirect pathway, urbanisation is the dependent variable and market access (distance to the nearest market, and distance to the nearest road) is the regressor. This analysis stems from an understanding of the nexus between market access and household food security (Usman and Haile 2022; Stifel and Minten 2017). There is a negative association between urbanisation and market access. In particular, urbanisation reduces distance to market and distance to road, which can be correlated with household food security.

**Table 7:** Mechanisms of the effects of urbanisation on food security.

Panel A	HFIAS score	FCS	HDDS
Ln (night‐time light)	−0.121*** (0.032)	2.877*** (0.257)	0.364*** (0.022)
Ln distance market	−0.213*** (0.041)	−0.591* (0.319)	−0.080*** (0.027)
Ln distance nearest road	−0.266*** (0.034)	−0.408 (0.293)	−0.072*** (0.025)
Constant	11.670*** (0.201)	50.574 (1.453)	8.122*** (0.125)

Panel B	Ln (night‐time light)	Ln (night‐time light)	Ln (night‐time light)
Ln distance market	−0.574*** (0.018)	−0.384*** (0.016)	−0.384*** (0.016)
Ln distance nearest road	−0.431*** (0.015)	−0.352*** (0.014)	−0.352*** (0.014)
Constant	4.153*** (0.072)	3.190*** (0.063)	3.190*** (0.063)

*Note:* *, **, and *** indicate levels of significance at 10%, 5%, and 1%. No control variables used. Both distance to the nearest road and distance to the nearest market are measured in kilometres.

Households located close to roads and markets are more likely to engaged in economic activities such as purchase and sell of commodities (agricultural commodities for rural households) compared to households located far away from roads and markets. Further, household proximity to markets and roads can be linked to access to varieties of food and food prices, which are associated with household food security (Usman and Haile 2022; Stifel and Minten 2017). However, we find counter‐intuitive results for the relationship between market access (both distance to market and distance to nearest road) and HFIAS score. It is important to note that the result of the nexus between HFIAS score and is not causal, hence, the correlation among the variables should be interpreted with caution. The counter‐intuitive results associated with HFIAS score may be due to possible confounding often associated with analysis based on correlation analysis.

## 5 Discussion

This study investigates the relationship between urbanisation and household food security in Nigeria, using night‐time lights as a proxy for urbanisation. In addition, we identified potential mechanisms or pathways through which urbanisation affects household food security.

First, we find that urbanisation is positively associated with household food security. However, except for the HFIAS score, the coefficients of night‐time lights squared have negative and significant effects on food security indicators, such as FCS and HDDS scores. These findings indicate significant nonlinearity in the relationship (association) between urbanisation (proxied by night‐time light) and household food security status. This nonlinearity suggests that there's an optimum level/threshold level of urbanisation beyond which food security is negatively affected. This finding relates to existing studies on urbanisation having significant effect on household welfare in Ethiopia (Abay et al. 2023). Second, we undertake further analysis by considering the nexus between urbanisation and disaggregated food categories or classes. We find that night‐time light intensity is positively associated and statistically significant with the consumption of cereals/grains, pulses/nuts, vegetables, meat/products, fats/oil, meat and meat product and sugar/sugar products, but not statistically significant with roots/tubers. However, we find a negative and statistically significant association between nightlight intensity squared and food classes such as cereals, roots/tubers, pulses, vegetables, meat, fats/oil, but not statistically significant with fruits, milk and sugar products.

Third, we identify potential pathways through which urbanisation affect household food security. Consistent with existing studies, market access captured as distance to the nearest market, and distance to the nearest road, is one of the plausible mechanisms through which urbanisation affect household food security (Usman and Haile 2022; Stifel and Minten 2017). Using SEM, we show how urbanisation affect market access, and consequently household food security. Urbanisation is likely to result in improved market access (a decline in distance to the nearest market and road), in turn, market access impacts food security.

This paper is not without limitations. First, although the findings of this paper reveal patterns of association between urbanisation and food security, the relationship between urbanisation and food security is not causal. This is due to the possibility of endogeneity associated with urbanisation. In other words, unobserved or omitted variables which could influence or affect urbanisation can also be correlated with food security. Nevertheless, the findings of our paper provide interesting insights into the correlation between urban growth and food security. The second limitation of this paper relates to lack of disaggregation of urban settlement into slums in urban or slums in rural areas using nighttime light. We identify that the disaggregation of urban settlement will make an interesting line for future research.

Second, despite the increasing recognition of the use of the DMSP night‐time light intensity to proxy the levels and dynamics of urbanisation, there are limitations associated with the use of these data (see Donaldson and Storeygard 2016; Michalopoulos and Papaioannou 2018). The Defense Meteorological Satellite Programme (DMSP) night‐light‐intensity data do not distinguish between luminosity caused by human activities and persistent lights produced by activities such as gas production (Elvidge et al. 2009). Also, the differences in light intensity caused by the variations in infrastructure (e.g., factories and transportation hubs) are not distinguished by the night‐time luminosity data. This could lead to the data not accurately capturing the levels of urbanisation in areas where artificial lights and gas flaring may be common (Elvidge et al. 2009). However, in our analysis, the test of the mean difference and Figure 4A reveal significant differences between the night‐time lights in rural and urban areas in Nigeria—with higher night‐time lights in urban areas than the rural areas. Thus, these luminosity data provide more variations and dynamics than the rural‐urban indicator used in previous studies.

Third, another key limitation in using night‐time light as a proxy for urbanisation is the lag between the establishment of human settlement and the provision of public utilities such as electricity. Unlike in the high‐income countries where the establishment of utilities often precedes home construction and occupancy, the reverse is the case in most low‐ to medium countries such as Nigeria. The intervening period between house occupancy to connection to the grid may range from months to a couple of years. Thus, remote sensing night‐time data may not always capture urban expansion in these jurisdictions in near‐real time. Nonetheless, in Nigeria this situation is mitigated in part by the widespread household provisioned electricity through the use of electric generators.

## 6 Conclusion

Household food insecurity is prevalent in many developing countries, with the extent or incidence of food insecurity possibly varying across the urban spectrum. In this study, we explore the variation in urbanisation across households over time to examine the implications of rapid urbanisation on households' food security in Nigeria. To measure urbanisation, we used satellite‐based night‐time light intensity data as a proxy for different stages or degrees of urbanisation and related economic activities. This study finds that urban expansion is associated with improved food security status across the different stages or thresholds of urbanisation, but higher urban expansion imperils food security.

In particular, the results of this study show that night‐time light is positively associated with household FCS and HDDS. However, we find that higher polynomial terms of night‐time light intensity exhibit a nonlinear relationship between urbanisation and household food security. Further, we identify heterogeneous effects on rural‐urban location, as well as the pathways through which urbanisation is associated with household food security in Nigeria. These results provide important insights into how urban expansion or growth could change the food security status of households in developing countries, and, precisely, in the Nigerian context.

The results of our paper reinforce the growing literature on the connection between urban growth and household well‐being. Hence, the findings are consistent with previous studies on the nexus between urbanisation and household welfare in developing countries (Abay et al. 2023; Calì and Menon 2013; Christiaensen and Todo 2014; Christiansen and Kanbur 2017; Gibson et al. 2017; and Ingelaere et al. 2018; Lanjouw and Marra 2018). For example, Lanjouw and Marra (2018) estimate welfare outcomes at the level of individual towns and cities in Vietnam, and the results show that households in larger cities have lower subjective welfare than households that are not in larger cities. Similarly, in an analysis of a sample of Indian districts from 1983 to 1999, Calì and Menon (2013) find that urbanisation has a significant poverty‐reducing effect, and the results are driven by increased demand for local agricultural products.

Overall, our paper contributes to the literature by providing new evidence using night‐time light as a measure of urban expansion and investigating its relationship with household food security in Nigeria. It is important to note that, to understand how urban expansion affect food security, market access, such as proximity to markets and roads, should be taken into consideration. Therefore, our results suggest that policies to improve household well‐being should consider market access as a plausible pathway towards achieving household food security in low‐ and middle‐income countries. Achieving household food security is connected to reduction in hunger, poverty, and improvement in well‐being, which have implications on SDG1 and SDG2 of the SDGs. Moreover, ensuring that household are food‐secure is critical in combating maternal and child malnutrition which is rife in many underserved communities of the low‐ and mid‐income countries.

## Author Contributions

Joseph B. Ajefu, Michael Henry, and Sabastine U. Ugbaje performed the research. Joseph B. Ajefu and Michael Henry designed the study and identified contributions of the literature. Joseph B. Ajefu and Sabastine U. Ugbaje analysed the data. Joseph B. Ajefu, Michael Henry, and Sabastine U. Ugbaje prepared the manuscript. All the authors were involved in reviewing, editing and approving the final manuscript for submission to the journal.

## Conflicts of Interest

The authors declare no conflicts of interest.

## Data Availability

The data set and the replication codes are available upon request through the corresponding author.

## Endnotes

1 Urbanisation refers to an “increase in the proportion of a population living in urban areas” and the “process by which a large number of people become permanently concentrated in relatively small areas, forming cities” (Organisation for Economic Co‐operation and Development 2012). It also connotes the shift from a population that is scattered across small rural settlement in which agriculture is the mainstay economic activity towards population that is concentrated in larger, dense urban settlement, in which development of manufacturing is the main driver and consequently results in urban agglomeration and employment growth (Montgomery et al. 2003). However, the patterns of urbanisation in many natural resource‐dependent countries, e.g., in Nigeria, rapid urbanisation occurs without industrialisation or manufacturing, which leads to growth of cities dominated by employment in non‐tradable services (Gollin et al. 2016).

2 Szabo (2016) uses country‐level data to examine the effect of urbanisation on food security. The results confirm a significant negative effect of urban expansion on food security at the country level.

3 Food security exists when “all people, at all times, have physical, social and economic access to sufficient, safe and nutritious food, which meets their dietary needs and food preferences for an active and healthy life” (FAO 1996).

4 Except for studies such as Van de Poel et al. (2009) and Van de Poel et al. (2012), which construct and use a continuous urbanicity index that takes into consideration community‐based characteristics, this index ranks communities or districts based on various characteristics that are related to urbanisation.

5 Night lights intensity data have been used to measure urbanisation by Keiser et al. (2004), Burchfield et al. (2006), Zhang and Seto (2011), and Ameye and De Weerdt (2019). Ameye and De Weerdt (2019) assert that using night‐time lights data as a proxy for urbanisation has the following advantages over other measures: (i) night‐time lights provide a plausible and continuous measure of the extent of urbanisation and related economic activity (ii) the definition of urbanisation provided is consistent across countries (iii) night‐time lights show variation within urban areas with regard to the degree of nightlight emitted.

6 See https://eogdata.mines.edu/products/dmsp.

7 Preprocessing of the data removes lights not associated with electricity. The excluded light sources include sunlit illumination; glare based on solar elevation angle; moonlit data; clouds affected observations; lighting features from the aurora in the northern hemisphere; and lights from ephemeral events such as fires.

8 See https://eogdata.mines.edu/products/dmsp.

9 Food consumption score is computed: (2 * number of days of grains)) + (2*number of days roots and tubers) + (3 * number of days of nuts and pulses) + (number of days of vegetables) + (4 * number of days of meat, fish, other meat, and eggs) + (number of days of fruits) + (4 * number of days of milk products) + (0.5 * number of days of fats and oils) + (0.5 * number of days of sugar, sugar products, and honey). Spices and condiments are excluded. It has a maximum value of 126.

10 The wave 4 (2018) of the Nigerian GHS does not contain information to compute the HFIAS score.

## 1

See Table A1.

**Table A1:** Urbanisation and Food Security Using Urban Dummy.

	HFIAS score	FCS	HDD
	(1)	(2)	(3)
Urban dummy	−0.123 (0.148)	5.828*** (1.692)	0.776*** (0.165)
Constant	6.263*** (2.044)	51.304* (27.319)	6.457*** (1.941)
Household control	Yes	Yes	Yes
Community control	Yes	Yes	Yes
Region fixed effect	Yes	Yes	Yes
Year fixed effect	Yes	Yes	Yes
R‐squared	0.207	0.071	0.154
Observations	3852	5392	5392

*Note:* *, **, and *** indicate levels of significance at 10%, 5%, and 1%. Additional control variables used are the same as reported in Table 3.
